# Protein Sub-Nuclear Localization Prediction Using SVM and Pfam Domain Information

**DOI:** 10.1371/journal.pone.0098345

**Published:** 2014-06-04

**Authors:** Ravindra Kumar, Sohni Jain, Bandana Kumari, Manish Kumar

**Affiliations:** Department of Biophysics, University of Delhi South Campus, New Delhi, India; CSIR-Institute of Microbial Technology, India

## Abstract

The nucleus is the largest and the highly organized organelle of eukaryotic cells. Within nucleus exist a number of pseudo-compartments, which are not separated by any membrane, yet each of them contains only a specific set of proteins. Understanding protein sub-nuclear localization can hence be an important step towards understanding biological functions of the nucleus. Here we have described a method, SubNucPred developed by us for predicting the sub-nuclear localization of proteins. This method predicts protein localization for 10 different sub-nuclear locations sequentially by combining presence or absence of unique Pfam domain and amino acid composition based SVM model. The prediction accuracy during leave-one-out cross-validation for centromeric proteins was 85.05%, for chromosomal proteins 76.85%, for nuclear speckle proteins 81.27%, for nucleolar proteins 81.79%, for nuclear envelope proteins 79.37%, for nuclear matrix proteins 77.78%, for nucleoplasm proteins 76.98%, for nuclear pore complex proteins 88.89%, for PML body proteins 75.40% and for telomeric proteins it was 83.33%. Comparison with other reported methods showed that SubNucPred performs better than existing methods. A web-server for predicting protein sub-nuclear localization named SubNucPred has been established at http://14.139.227.92/mkumar/subnucpred/. Standalone version of SubNucPred can also be downloaded from the web-server.

## Introduction

Nuclear proteins are produced in cytoplasm from where they are transported to the nucleus. Unlike other compartmentalized organelles such as mitochondria and chloroplast, no membrane bound sub-nuclear partition exists inside the nucleus. Even then, every nuclear protein localizes to its specific location within the nucleus forming a number of virtual sub-nuclear compartments like nucleolus, nuclear matrix, centromere etc. At present several experimental methods like co-expression of fluorescent proteins [1], electron and fluorescence microscopy [2], [3], immuno-fluorescence labeling [4], [5], photo-activated localization microscopy [6], liquid-chromatography-tandem mass spectrometry [7], [8] etc are available to study protein localization. But the requirement of time and resources limit their usage.

Localization of a protein strongly correlates with its function. Thus understanding the subcellular localization of a protein can be of fundamental importance in revealing different regulatory mechanism. For example, alterations in gene expression of proteins located in different sub-nuclear locations may cause cancer and other genetic diseases [9], [10]. Therefore knowledge of proteins sub-nuclear localization is essential not only for understanding the cellular processes and genomic regulation but also to understand the clinico-pathological manifestations caused due to mis-localized nuclear proteins. The prediction of protein localization at the sub-nuclear level is difficult compared to the generalized subcellular level due to (i) absence of physical barrier or membrane within the cell nucleus [11] and (ii) dynamic nature of protein complexes within the nucleus [12].

In the past, several attempts have been made to predict the sub-nuclear localization of nuclear proteins [13]–[21]. Shen and Chou [13] developed a pseudo amino acid composition based Optimized Evidence-Theoretic K-nearest classifier to predict proteins at 9 different sub-nuclear locations viz. Cajal body, chromatin, heterochromatin, nuclear diffuse, nuclear pore, nuclear speckle, nucleolus, PcG body and PML body. Lei and Dai [14] used Support Vector Machine (SVM) for prediction of six sub-nuclear classes (PML body, nuclear lamina, nuclear splicing speckles, chromatin, nucleoplasm and nucleolus). Huang et al [15] proposed an evolutionary support vector machine based classifier, ProLoc, trained on a large set of physicochemical composition features using dataset of [13] and [14]. Mundra et al [16] reported a multi-class SVM based classifier using amino acid attributes, dipeptide composition, pseudo amino acid composition and PSSM on dataset of [13] and reported better performance. Shen and Chou [17] again developed a 9 sub-nuclear location (chromatin, heterochromatin, nuclear envelope, nuclear matrix, nuclear pore complex (NPC), nuclear speckle, nucleolus, nucleoplasm and nuclear PML body) predictor, Nuc-PLoc, using combination of evolutionary information and pseudo-amino acid composition. Li and Li [18] proposed another method using an algorithm of increment of diversity combined with improved quadratic discriminant analysis by using amino acid and pseudo amino acid compositions. Jiang et al [19] reported an ensemble classification method for sub-nuclear locations on dataset of [13] and [14] using decision trumps, Fuzzy K-Nearest Neighbors algorithm and radial basis-SVMs.

All the sub-nuclear protein localization methods reported above have following problems which need to be taken care of: (1) the datasets used were SNL9 [13] (in case of [15], [16], [19]), SNL6 [14] (in case of [15], [18]–[21] and Nuc-PLoc [17] in case of [20], [21]. These datasets (SNL6, SNL9 and Nuc-PLoc dataset) were collected more than five years ago and our analysis revealed that in current SwissProt annotations, their locations have changed in the intervening period. (2) Highly homologous sequences were included in their benchmark datasets because the cutoff to remove homologous sequences in SNL6 [14] and Nuc-PLoc dataset [17] is <50% and ≤80% respectively while in case of SNL9 only one criteria was used i.e. ‘protein sequences having same name but from different species, only one of them was included to reduce the redundancy’ [13]. Therefore a more stringent non-redundant dataset is needed to avoid homology bias during training and also to include the recent progress in the field of protein function annotation.

In this paper, we report a method for prediction of sub-nuclear localization of proteins using two different approaches, namely Method-I and Method-II. At start of a prediction cycle, the query sequence is presented to Method-I, which checks the presence or absence of unique Pfam domains and if it does not find any unique domain prediction is referred to Method-II, which is based on amino acid composition based SVM model. We considered the following 10 sub-nuclear locations for the present study: (i) centromere, (ii) chromosome, (iii) nuclear speckle, (iv) nucleolus, (v) nuclear envelope, (vi) nuclear matrix, (vii) nucleoplasm, (viii) nuclear pore complex (ix) PML body and (x) telomere. First we evaluated the upper limit of the prediction on the basis of presence of location specific Pfam domains that were identified by Hidden Morkov model and SVM model separately. In order to exploit the advantages of both the approaches, we used both methods sequentially and obtained higher prediction accuracy. The final method thus developed, was called ‘SubNucPred’. We also benchmarked performance of SubNucPred vis-à-vis other existing methods on an independent dataset. A web server as well as standalone package was also established at http://14.139.227.92/mkumar/subnucpred/ to make SubNucPred available for public usage.

## Materials and Methods

### Datasets

#### Training Dataset (Data^MAIN^)

Protein sequences used in this work were obtained from the SwissProt protein database (version 94.0). We applied the following qualifiers to obtain high-quality protein sequences: (i) location experimentally confirmed and limited to a single sub-nuclear location, (ii) a full-length protein, (iii) protein length between 50–3000 amino acids, (iv) subcellular location annotation should not contain the term ‘probable’, ‘potential’ or ‘by similarity’ (v) should not contain membranous proteins and (vi) protein existence must be proven experimentally. Using the above-mentioned criteria, 1000 nuclear proteins belonging to 10 different sub-nuclear locations (nucleolus, chromosome, centromere, nuclear speckle, telomere, nucleoplasm, nuclear matrix, nuclear envelope, nuclear pore complex, PML body) were obtained. In order to ensure that each location had sufficient number of proteins, only locations having more than 10 sequences were considered. As SwissProt contains a lot of redundant proteins, which may result in over-estimation of prediction capability, we reduced the redundancy among sequences at 40% using CD-HIT [22] and obtained 669 proteins (Table S1). We also added 100 randomly selected non-redundant non-nuclear proteins to provide comprehensive information about the protein universe to enable discrimination between nuclear and non-nuclear proteins.

As described earlier, three datasets namely SNL6, SNL9 and Nuc-PLoc dataset were mainly used for developing and benchmarking other sub-nuclear prediction methods. In SNL6 following 6 locations were considered: PML body, nuclear lamina, nuclear splicing speckles, chromatin, nucleoplasm and nucleolus while in SNL9 the locations considered were PML body, nuclear speckle, chromatin, nuclear diffuse, nucleolus, Cajal body, heterochromatin, nuclear pore and PcG body. Nuc-PLoc dataset comprised nuclear proteins in 9 sub-nuclear locations i.e., chromatin, heterochromatin, nuclear envelope, nuclear matrix, nuclear pore complex, nuclear speckle, nucleolus, nucleoplasm and nuclear PML body. In SubNucPred we tried to include as many locations as possible. For example we have included all SNL6 locations except nuclear lamina (due to retrieval of less than 10 sequences). If we compare the SNL9 locations vis-à-vis SubNucPred, five locations (nuclear speckle, nucleolus, nuclear pore, nuclear diffuse/nucleoplasm and PML body) are common in both. Cajal body and PcG body was not considered due to presence of less than 10 sequences. Further in SubNucPred rather than simply categorizing a protein as chromatin or heterochromatin (as was done in SNL9 and Nuc-PLoc dataset) we have assigned precise locations such as chromosome, centromere and telomere.

#### Benchmarking Dataset (Data^IND^)

For benchmarking our method we downloaded all nuclear proteins of the above ten locations from SwissProt (version 113), which were not present in training dataset using the retrieval criteria of Data^MAIN^. While compiling this dataset, we made sure that no sequence of Data^IND^ had homologous sequence in Data^MAIN^. Further no two sequences of Data^IND^ have more than 40% identity with each other. We found 31 sequences in centromere, 38 in chromosome, 14 in nuclear speckle, 46 in nucleolus, 51 in nuclear envelope, 6 in nuclear matrix, 7 in nucleoplasm, 2 in nuclear pore complex, 7 in PML body and 5 in telomere (Table S1).

### Unique Single Sub-nuclear Location Domain Library

Pfam is a database of protein domain families in which each family is represented by multiple sequence alignments and profile hidden markov models (HMM) [23]. The idea behind using Pfam domain is the fact that in eukaryotes, specialized functions is carried out at specific locations only. It means that domain which is exclusively found in a specific location may be used to carry out the localization prediction.

In Pfam each HMM represents one Pfam domain. We used manually curated section of Pfam, Pfam-A, (version-26.0) containing 13,672 families. 1,000 redundant proteins, retrieved from SwissProt for constructing Data^MAIN^, were searched against the Pfam database using ‘hmmpfam’ model of HMMER package [24] at an E-value threshold of 1e−5. Redundant proteins were preferred over entire Data^MAIN^ because it has been reported by Guda et al [25] that clustering at sequence identities lower than 90% produce smaller and diverse data sets, resulting in loss of some unique Pfam domains. After searching the Pfam domain library, we found 384 domains in total, which were present only in single sub-nuclear location (Table S2).

Since the unique domain library was compiled using nuclear proteins only, it might be possible that some of the 384 domains may be present in extra nuclear locations. In order to make an unambiguous domain library, we downloaded 90% redundancy reduced non-nuclear proteins (65,076 proteins) from SwissProt. The criteria used in downloading were same as in Data^MAIN^ but here we considered membrane proteins also to avoid loss of even a single non-nuclear domain. HMMER found a total of 7,879 Pfam domains in them at E-value threshold 1e−5. After removing all domains that were common in both nuclear and non-nuclear proteins, only 171 unique domains (Table S2) were obtained which were present in a single sub-nuclear location, henceforth called as single sub-nuclear location domain (SSLD). SSLDs are exclusively found in a single sub-nuclear location and nowhere else, neither at remaining nuclear nor non-nuclear locations (names of all SSLD are listed in Table S3).

We also tried to find out the domains, which were unique for more than one sub-nuclear location and got two such domains for locations ‘centromere and chromosome’ and ‘centromere and PML body’. We found one unique domain for ‘chromosome and nucleoplasm’, ‘chromosome and telomere’, ‘nuclear envelope and nuclear pore complex’, ‘nuclear speckle and nuclear matrix’ and ‘nuclear matrix and nucleolus’ (Table S4). We could not find domains unique for more than two different locations. This shows that different sub-nuclear locations share very few domains, which may be due to very specific functional nature of nuclear proteins.

### Support Vector Machine

SVM is one of the most common machine learning algorithm used for development of several bioinformatics prediction methods [15], [26]–[33]. SVM takes a set of feature vector attributes along with their real output as input. During training, SVM maps the input space into higher dimensional feature space thereby separating a given set of labelled data with an optimal hyperplane. As a result of training, SVM generates a model which can be used for the prediction of unknown examples. A detailed description of SVM can be obtained from Vapnik [34]. In this work, SVM_light software was employed to perform the prediction (available at http://svmlight. joachims.org).

### Cross-Validation and Performance Evaluation

In the present study, we adopted one-against-all approach to develop trained SVM models. It constructs i SVM models, where i is the number of classes. The i-th SVM is trained on all examples of i-th class with positive labels and all examples of remaining classes with negative labels. Hence during a complete training cycle, at once, example of each location is labeled positive. Same approach has been used earlier for this type of problem [14]. While training we adopted jack-knife/leave-one-out cross-validation (LOOCV) approach. Although LOOCV is time consuming, it is considered better than other methods of cross-validation [35]–[44]. During the LOOCV, each protein in the dataset was, in turn, singled out for testing by the classifier trained with the remaining proteins. To evaluate the performance, we calculated following indices for each complete cycle of LOOCV: true positive (TP), true negative (TN), false positive (FP), false negative (FN), sensitivity, specificity, accuracy and Matthews Correlation Coefficient (MCC), as formulated below:
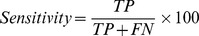


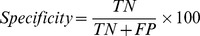


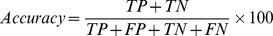


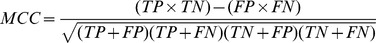



Where TP and TN were number of proteins whose location was correctly predicted while FP and FN were number of proteins whose location was wrongly predicted.

### Prediction Schema

The objective of the present work was to develop a prediction method that can predict the sub-nuclear location of a protein. The proposed predictor first searches for Pfam domains in the query sequence. If a Pfam domain is found, the predictor directs the query sequence to Method-I where the location is predicted on the basis of SSLD. If Method-I does not find any SSLD, the query is forwarded to Method-II, which uses amino acid composition based SVM modules for prediction. The procedure adopted by Method-II is an example of multi-class classification because we were trying to predict one among many candidate classes. A simple strategy to handle multi-class classification is to divide whole problem into a series of binary classifications and develop predictor for each class. This is popularly known as “one-versus-rest” approach [45], [46]. During development of each binary classifier, one class is considered as positive while the remaining classes as negative. In this work for each binary classification, one sub-nuclear location was treated as positive while remaining all classes as negative. It means an SVM trained to predict proteins of a particular sub-nuclear location was trained with all samples of that location with positive label and proteins of remaining locations with negative label. The same approach has been used in a number of earlier studies like prediction of subcellular localization [47], [48], G-protein coupled receptors [49]–[52], NRP protein sub-family prediction [53]–[56].

**Figure 1:**
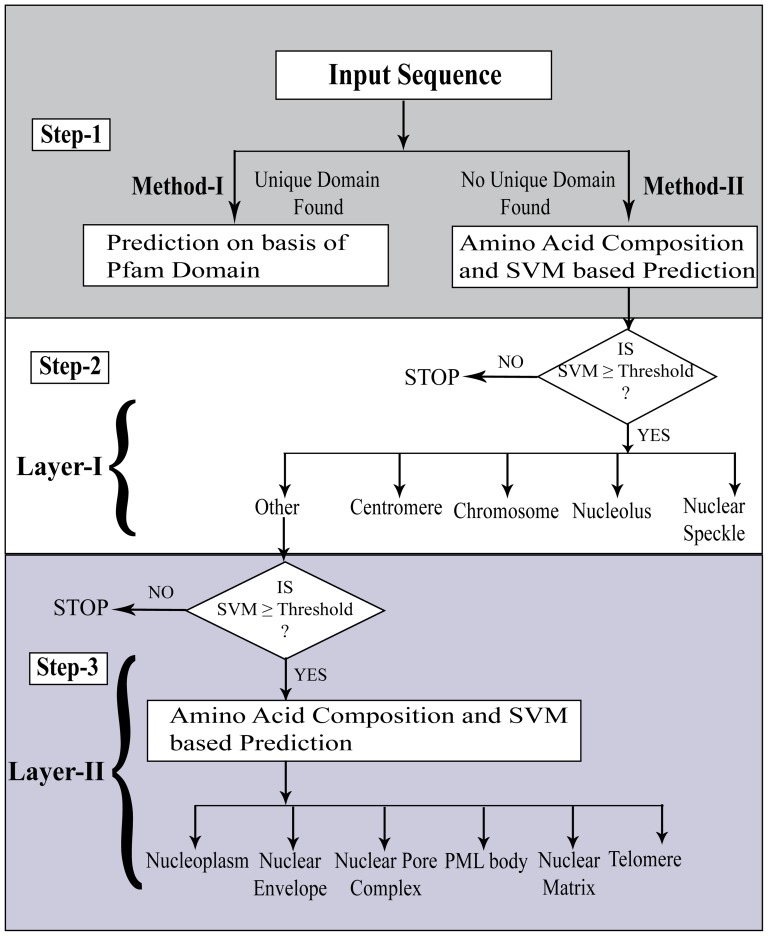
Flow diagram of SubNucPred. The overall schema is divided into three steps. Step-1 does prediction on the basis of presence or absence of unique Pfam domain (Method-I). Step-2 and 3 (referred as Layer-I & II respectively in manuscript) does prediction on the basis of amino acid composition based SVM model and threshold (Method-II). In step 2 or Layer-I, prediction is made for five sub-nuclear locations (centromere, chromosome, nucleolus, nuclear speckle and others). In case the SVM score of location ‘others’ is greater than the threshold, query protein is predicted to belong to locations contained in ‘Others’. 3rd step or Layer-II SVM prediction is used then and prediction is also done for the six locations belonging to ‘Others’.

When the “one-versus-rest” method is applied, the data imbalance problem will emerge since positive examples tend to be under-represented in comparison to the larger number of proteins of other classes (negative examples). As evident in Table S1, the dataset is heavily imbalanced with the ratio of positive to negative class protein varying around 1∶1 to 1∶55. A classifier based on an imbalanced data, will usually be biased towards the majority class, which can reduce the accuracy for the dataset having smaller number of samples or of less diversity. Thus there is a danger that the dominant data points may overwhelm the information provided by the less abundant data points. In order to overcome this problem, we adopted two-layered training approach. In first layer we kept location with more than 50 proteins viz (1) centromere (2) chromosome (3) nuclear speckle (4) nucleolus and (5) others containing all locations having less than 50 proteins (nuclear envelope, nuclear matrix, nucleoplasm, nuclear pore complex, PML body and telomere). In second layer, sequences belonging to locations ‘others’ were used only and SVM models were developed using the data of ‘others’ location only. This artifical division among different sub-nuclear locations enabled us to reduce the ratio of Positive/Negative data with 1∶1 to 1∶12 at layer-I and 1∶2 to 1∶9 at layer-II. In short, SubNucPred presents the query protein to layer-I and predicts it as a member of class whose SVM prediction score is equals to or greater than the threshold. The algorithm will enter into layer-II only if the SVM score of others is equal to or more than the threshold of prediction (Figure 1).

### Calculation of Protein Features

#### Amino Acid Composition

Amino acid composition is a quantitative measurement of each amino acid within a sequence. Following equation was used to compute the amino acid composition.
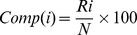



Here *Comp(i)* is amino acid composition of residue type *R_i_* and *N* is the total number of amino acids in particular protein.

#### Dipeptide Composition

In order to understand the compositional biasness, we also calculated the dipeptide composition. It gives information about the fraction of amino acid as well as their local order by a fixed length pattern of 400 possible dipeptides.

Following equation was used to compute the dipeptide composition.
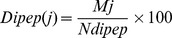



Here *Dipep(j)* is dipeptide composition of dipeptide type *M_j_*, where *j* can be any of the 400 dipeptides and *N_dipep_* is the total number of possible dipeptide in the protein.

#### Physicochemical Properties

Han et al [21] has reported a sub-nuclear localization prediction method using a set of 30 physicochemical properties obtained from AAindex database [57]. In this work we used the same properties to train the SVM. The input vector for physicochemical properties based SVM was created by multiplying the value of each physicochemical property with the corresponding amino acid composition. Therefore for each protein the dimension of input vector would be 20×30 = 600. The list of physicochemical property used in this work is provided in Table S5.

## Results and Discussion

### Amino acid Composition Analysis

**Figure 2:**
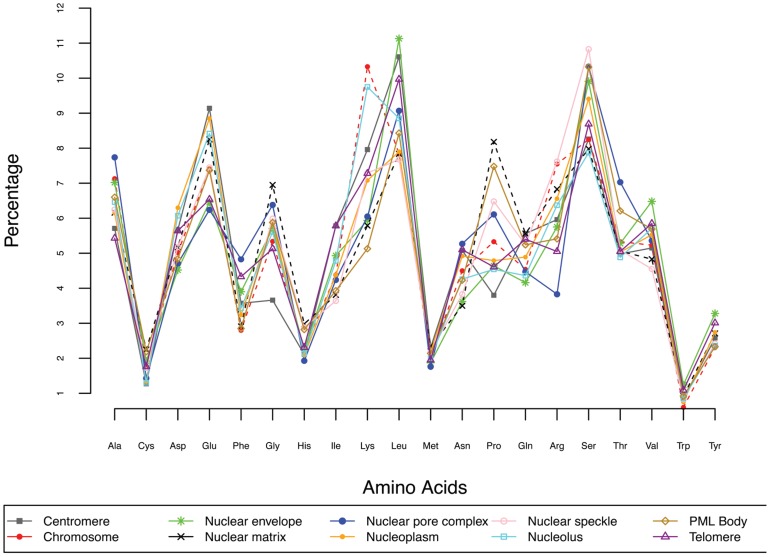
Average amino acid composition analysis of proteins belonging to different sub-nuclear locations.

We calculated the average amino acid composition of different sub-nuclear locations (Figure 2) and performed ANOVA test to find out statistically significant difference in amino acid compositions of different sub-nuclear locations. The test showed that at P-value 0.01 all except sulphur containing amino acid (Cys and Met) had statistically significant difference in amino acid composition (Table S6). We also observed that in a group of amino acids having same physicochemical property, composition of few amino acids had more variations. For example, variability in occurrence of Glu and Lys in different sub-nuclear locations is more than what is observed in other amino acids having similar property i.e. Asp and Arg respectively. Amino acids whose presence has significant influence on protein conformation, namely Gly and Pro also appeared to vary considerably in different sub-nuclear locations. Sulphur containing (Cys and Met) and aromatic amino acids (Phe, Trp and Tyr) were less abundant and showed very little variation among all the sub-nuclear locations.

### HMM Based Searching of SSLD

In order to estimate the number of proteins whose location can be predicted using the presence of unique domain(s) only, we searched the presence of SSLD in the proteins of Data^MAIN^ (referred as Method-I). We were able to correctly predict only 45.35%, 19.47%, 24.00%, 34.69%, 41.18%, 27.78%, 20.00%, 25.00%, 25.00% and 27.03% proteins belonging to centromere, chromosome, nuclear speckle, nucleolus, nuclear envelope, nuclear matrix, nucleoplasm, nuclear pore complex, PML body and telomere respectively (Table 1). It shows that presence of location specific domain(s) is not sufficient to predict even half of their proteins. The result also indicates that very few sub-nuclear specific domains are present in the nuclear proteins and these nuclear proteins are composed of a limited set of domains.

**Table 1:** Prediction efficiency at various sub-nuclear locations on the basis of presence of SSLD.

Location	Number of Unique Domains	Proteins Predicted	Prediction Efficiency (%)
Centromere (86)	21	39	45.35
Chromosome (113)	19	22	19.47
Nuclear speckle (50)	16	12	24.00
Nucleolus (294)	81	102	34.69
Nuclear envelope (17)	6	7	41.18
Nuclear matrix (18)	3	5	27.78
Nucleoplasm (30)	7	6	20.00
Nuclear pore complex (12)	4	3	25.00
PML body (12)	4	3	25.00
Telomere (37)	10	10	27.03

SSLD represents single sub-nuclear domain.

Values in parenthesis are the number of proteins in that location.

### SVM Modules

#### Amino Acid Composition Based SVM

Using one-versus-rest and LOOCV approach of training we found 76.85% accuracy for centromere, 64.89% for chromosome, 72.17% for nuclear speckle, 72.43% for nucleolus, 69.05% for nuclear envelope, 67.62% for nuclear matrix, 58.65% for nucleoplasm, 77.24% for nuclear pore complex, 62.55% for PML body and 68.14% for telomere (Table S7 and Table S8).

#### Amino Acid Composition Based SVM with Two-Layered Training Approach

As evident in Table S7, performance of SVM was poor for locations having less number of proteins namely, nuclear envelope, nuclear matrix, nucleoplasm, nuclear pore complex, PML body and telomere. The reduced performance might have occurred due to the highly skewed nature of positive/negative example ratio.

In order to reduce the unbalancing of data, we reorganized whole data in two groups and adopted two-layered training approach. In the first layer, we included locations having more than 50 proteins viz. centromere, chromosome, nuclear speckle, nucleolus and others (all locations having less than 50 proteins viz. nuclear envelope, nuclear matrix, nucleoplasm, nuclear pore complex, PML body and telomere). In second layer sequences belonging to locations merged in class ‘Others’ of first layer were only used. With layered approach we observed sharp increase in overall performance. At first layer, we obtained overall accuracy 76.85%, 64.89%, 73.08%, 71.91% and 68.14% respectively for centromere, chromosome, nuclear speckles, nucleolus, and ‘others’ location (Table 2 and Table S9). At the second layer, we obtained overall accuracy 75.40%, 69.84%, 67.46%, 78.57%, 66.67% and 72.22% for nuclear envelope, nuclear matrix, nucleoplasm, nuclear pore complex, PML body and telomere respectively (Table 2 and Table S9).

**Table 2:** Performance of SVM model based on amino acid composition using layer approach. (For detail please see Table S9).

Location	TP	TN	FP	FN	Sensitivity	Specificity	Accuracy	MCC	AUC
**Layer-I**									
Centromere (86)	67(44)	524(628)	159(55)	19(42)	77.91(51.16)	76.72(91.95)	76.85(87.39)	0.38(0.41)	0.83
Chromosome (113)	76(38)	423(606)	233(50)	37(75)	67.26(33.63)	64.48(92.38)	64.89(83.75)	0.23(0.29)	0.71
Nuclear speckle (50)	35(15)	527(701)	192(18)	15(35)	70.00(30.00)	73.30(97.50)	73.08(93.11)	0.23(0.33)	0.80
Nucleolus (294)	211(162)	342(411)	133(64)	83(132)	71.77(55.10)	72.00(86.53)	71.91(74.51)	0.43(0.44)	0.78
Others (126)	86(86)	438(438)	205(205)	40(40)	68.25(68.25)	68.12(68.12)	68.14(68.14)	0.28(0.28)	0.72
**Layer-II**									
Nuclear envelope (17)	12(8)	83(100)	26(9)	5(9)	70.59(47.06)	76.15(91.74)	75.40(85.71)	0.35(0.39)	0.76
Nuclear matrix (18)	13(5)	75(104)	33(4)	5(13)	72.22(27.78)	69.44(96.30)	69.84(86.51)	0.30(0.33)	0.72
Nucleoplasm (30)	20(23)	65(59)	31(37)	10(7)	66.67(76.67)	67.71(61.46)	67.46(65.08)	0.30(0.33)	0.67
Nuclear pore complex (12)	9(9)	90(90)	24(24)	3(3)	75.00(75.00)	78.95(78.95)	78.57(78.57)	0.36(0.36)	0.80
PML body (12)	8(8)	76(76)	38(38)	4(4)	66.67(66.67)	66.67(66.67)	66.67(66.67)	0.20(0.20)	0.66
Telomere (37)	27(20)	64(82)	25(7)	10(17)	72.97(54.05)	71.91(92.13)	72.22(80.95)	0.42(0.51)	0.76

Where TP, TN, FP, FN, MCC and AUC are True positive, True negative, False positive, False negative, Matthews correlation coefficient and Area under ROC curve respectively.

Values in parenthesis are the number of proteins in that location at column ‘location’ and in column ‘TP’, ‘TN’, ‘FP’, ‘FN’, ‘Sensitivity’, ‘Specificity’, ‘Accuracy’ and ‘MCC’ are the values at which maximum MCC was found.

As layered approach of training performed better, we adopted this in all other SVM modules described in following sections.

#### Dipeptide Composition Based SVM with Two-Layered Training Approach

In this work we also tried to use dipeptide composition based SVM model. In general, dipeptide composition based SVM model are better than amino acid composition based models [52], [58]–[60]. But in this study performance of dipeptide composition based SVM models was significantly poor than amino acid based SVM models. Here we found 76.72% accuracy for centromere, 69.44% for chromosome, 72.17% for nuclear speckle, 73.08% for nucleolus, 65.02% for others 70.63% for nuclear envelope, 59.52% for nuclear matrix, 61.11% for nucleoplasm, 68.25% for nuclear pore complex, 57.94% for PML body and 69.84% accuracy for telomere (Table S10).

#### Physicochemical Properties Based SVM with Two-Layered Training approach

At first layer, we obtained overall accuracy 73.60% for centromere, 57.22% for chromosome, 73.73% for nuclear speckle, 68.53% for nucleolus and 65.28% for others (Table S11). At the second layer, we found 57.14% accuracy for nuclear envelope, 60.32% for nuclear matrix, 64.29% for nucleoplasm, 77.78% for nuclear pore complex, 61.11% for PML body and 65.87% for telomere (Table S11).

It is clear from Table 2 and Tables S9-S11 that hierarchical approach of training with amino acid composition showed maximum performance and hence this model was used for further analysis. It is referred as Method-II henceforth.

### Receiver Operating Characteristics Curve and Area Under ROC Curve Analysis

**Figure 3:**
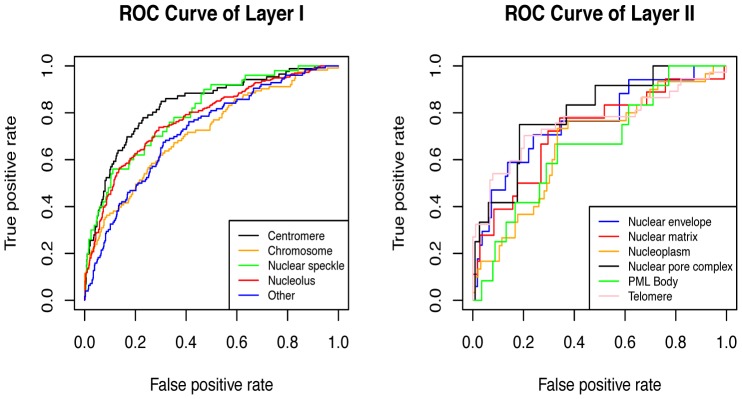
ROC curve of amino acid composition based SVM modules.

When a classifier has to do the multi-class classification, especially on an imbalanced dataset, as is the case with the present work, overall accuracy might be an unrealistic assessment of classifier's performance due to the correct classification to the classes in majority. Hence to avoid biasness, the prediction capability of SVM model was assessed at the threshold where sensitivity and specificity values are nearly equal. This also took care of the biasness in value of accuracy due to unequal number of positive and negative examples and enabled us to analyze the performance on the basis of overall accuracy. Another way of unbiased estimation of classifier's accuracy is by using the receiver operating characteristic (ROC) [61] plot, which is a very popular way of analyzing the overall performance of a classifier system. It shows the tradeoff between sensitivity and specificity at various thresholds and is created by plotting sensitivity (True positive rate) vs 1-specificity (False positive rate). The area under the ROC curve (AUC) is commonly used as a summary measure of diagnostic accuracy. The ROC plot (Figure 3, Figure S1 and Figure S2) and corresponding AUC values (Table 2, Table S10 and Table S11) clearly shows that amino acid composition based SVM modules can predict sub-nuclear localization at very high accuracy and was better than dipeptide composition and physicochemical properties based SVM modules.

### Combined Approach of Prediction

The above observations clearly show that neither the presence of Pfam domains nor SVM alone is sufficient to do prediction with very high accuracy. Hence we used both approaches sequentially to get advantages of them. In the current method, first and foremost Pfam domains were searched in a protein and if any SSLD was found, the protein was predicted to belong to that particular sub-nuclear location (Method-I). If a protein lacked any SSLD then we used SVM module for prediction (Method-II). By using this approach at layer-I prediction accuracy for centromere, chromosome, nuclear speckle, nucleolus and others were 85.05%, 76.85%, 81.27%, 81.79% and 82.05% respectively (Table 3). At layer-II, the prediction accuracy for nuclear envelope, nuclear matrix, nucleoplasm, nuclear pore complex, PML body and telomere were 79.37%, 77.78%, 76.98%, 88.89%, 75.40% and 83.33% respectively (Table 3). The results clearly show that this approach significantly increases the accuracy of sub-nuclear protein prediction. This new combined method has been named as SubNucPred.

**Table 3:** Performance of SSLD and amino acid composition based SVM.

Location	TP	TN	FP	FN	Sensitivity	Specificity	Accuracy	MCC
**Layer-I**								
Centromere	73(60)	581(653)	102(30)	13(26)	84.88(69.77)	85.07(95.61)	85.05(92.72)	0.53(0.64)
Chromosome	88(56)	503(623)	153(33)	25(57)	77.88(49.56)	76.68(94.97)	76.85(88.30)	0.42(0.49)
Nuclear speckle	39(23)	586(705)	133(14)	11(27)	78.00(46.00)	81.50(98.05)	81.27(94.67)	0.35(0.51)
Nucleolus	245(213)	384(435)	91(40)	49(81)	83.33(72.45)	80.84(91.58)	81.79(84.27)	0.63(0.66)
Others	95(95)	536(536)	107(107)	31(31)	75.40(75.40)	83.36(83.36)	82.05(82.05)	0.49(0.49)
**Layer-II**								
Nuclear envelope	13(12)	87(101)	22(8)	4(5)	76.47(70.59)	79.82(92.66)	79.37(89.68)	0.43(0.59)
Nuclear matrix	13(7)	85(104)	23(4)	5(11)	72.22(38.89)	78.70(96.30)	77.78(88.10)	0.39(0.44)
Nucleoplasm	22(24)	75(70)	21(26)	8(6)	73.33(80.00)	78.12(72.92)	76.98(74.60)	0.46(0.46)
Nuclear pore complex	11(11)	101(101)	13(13)	1(1)	91.67(91.67)	88.60(88.60)	88.89(88.89)	0.60(0.60)
PML body	9(9)	86(86)	28(28)	3(3)	75.00(75.00)	75.44(75.44)	75.40(75.40)	0.33(0.33)
Telomere	32(27)	73(83)	16(6)	5(10)	86.49(72.97)	82.02(93.26)	83.33(87.30)	0.64(0.69)

Where TP, TN, FP, FN and MCC are True positive, True negative, False positive, False negative and Matthews correlation coefficient respectively.

Values in parenthesis are the values at which maximum MCC was found at respective column.

### Benchmarking on Independent Dataset

We benchmarked SubNucPred on Data^IND^ and found 65.22% accuracy for centromere, 65.70% for chromosome, 67.15% for nuclear speckle, 76.33% for nucleolus, 60.26% for nuclear envelope, 73.08% for nuclear matrix, 64.10% for nucleoplasm, 70.51% for nuclear pore complex, 70.51% for PML body and 52.56% accuracy for telomere (Table 4). The confusion matrix generated by prediction on the basis of presence of unique domains only is shown in Table S12.

**Table 4:** Performance of SubNucPred method on DataIND (One-vs-Rest approach).

Location	TP	TN	FP	FN	Sensitivity	Specificity	Accuracy	MCC
**Layer-I**								
Centromere (31)	17	118	58	14	54.84	67.05	65.22	0.16
Chromosome (38)	25	111	58	13	65.79	65.68	65.70	0.25
Nuclear speckle (14)	12	127	66	2	85.71	65.80	67.15	0.27
Nucleolus (46)	31	127	34	15	67.39	78.88	76.33	0.41
Others (78)	60	73	56	18	76.92	56.59	64.25	0.33
**Layer-II**								
Nuclear envelope (51)	27	20	7	24	52.94	74.07	60.26	0.26
Nuclear matrix (6)	3	54	18	3	50.00	75.00	73.08	0.15
Nucleoplasm (7)	4	46	25	3	57.14	64.79	64.10	0.13
Nuclear pore complex (2)	1	54	22	1	50.00	71.05	70.51	0.07
PML body (7)	4	51	20	3	57.14	71.83	70.51	0.18
Telomere (5)	3	38	35	2	60.00	52.05	52.56	0.06

Where TP, TN, FP, FN and MCC are True positive, True negative, False positive, False negative and Matthews correlation coefficient respectively.

Values in parenthesis are the number of proteins in that location.

### Comparison of SubNucPred with Existing Web-Server

It is important to compare the performance of a newly developed method with the existing methods to justify the need and usage of new method. Among a number of sub-nuclear prediction methods reported earlier, we found only three have working web-interface namely sub-nuclear compartments prediction system (Scp) [14], Nuc-PLoc [17] and Snlpred [21]. Therefore we compared the performance of our method with Scp, Nuc-PLoc and Snlpred using Data^IND^ (Table 5).

**Table 5:** Comparison of performance of SubNucPred with Nuc-PLoc, Snlpred and Scp web-servers using Data^IND^.

Location	SubNucPred	Scp$	Nuc-PLoc&	Snlpred#
Centromere (31)	15	2 Chromatin	7 Chromatin + 2 Hetrochromatin	11 Chromatin
Chromosome (38)	16	1 Chromatin	2 Chromatin	7 Chromatin
Nuclear speckle (14)	12	4	3	5
Nucleolus (46)	35	10	43	37
Nuclear envelope (51)	31	47 Nuclear Lamina	18	7 Nuclear Lamina
Nuclear matrix (6)	2	0	1	0
Nucleoplasm (7)	1	0	0	1
Nuclear pore complex (2)	1	1 Nuclear Lamina	0	2 Nuclear Lamina
PML body (7)	2	0	1	0
Telomere (5)	2	0	1 Chromatin	1 Chromatin

As all Scp, Nuc-Ploc and Snlpred don't have same sub-nuclear locations as in SubNucPred, we adjusted prediction of centromeric, chromosomal and telomeric protein to chromatin and hetrochromatin as correct prediction. Similarly for nuclear envelope and nuclear pore complex a prediction saying nuclear lamina was classified as correct.

Values in parenthesis are the number of proteins in that location.

$ Lei Z, Dai Y (2005) An SVM-based system for predicting protein subnuclear localizations. BMC Bioinformatics 6: 291.

& Shen HB, Chou KC (2007) Nuc-PLoc: a new web-server for predicting protein subnuclear localization by fusing PseAA composition and PsePSSM. Protein Eng Des Sel 20: 561–567.

# Han GS, Yu ZG, Anh V, Krishnajith AP, Tian YC (2013) An ensemble method for predicting subnuclear localizations from primary protein structures. PLoS One 8: e57225.

In the SubNucPred web-server, prediction is done by comparing prediction scores of each model to the threshold of prediction. A query protein is assigned to the location(s), whose SVM prediction score is greater or equals to the threshold. The overall prediction schema of SubNucPred web-server prediction works as followings. The query protein first goes to the Method I. If a unique domain is found, the location is assigned on the basis of domain. In case, no unique domain is found, the query is forwarded to the Method II. If the SVM score is greater than the threshold, the query protein is assigned to the locations. In case of location ‘Others’ if the SVM score of location ‘others’ were greater than the threshold of prediction, the second layer prediction will be done.

Due to the difference in number and nature of sub-nuclear locations, it is not possible to do one by one comparison of performance of four web-servers. For the locations identical in all four web-servers i.e. nuclear speckle, nucleolus, nuclear matrix, nucleoplasm and PML body, except nucleolus, the performance of SubNucPred web-server is better (Table 5). In case of nucleolar proteins, SubNucPred performance was better than Scp but inferior than Nuc-Ploc and Snlpred. We also analyzed performance for locations which are not identical among all four web-servers i.e. centromere, chromosome, nuclear envelope, nuclear pore complex and telomere and found that, the performance of SubNucPred is better in case of centromere, chromosome and telomere. It was also observed that Scp, Nuc-Ploc and Snlpred did not predict centromeric protein as centromeric; Scp predicted 2 proteins as chromatin binding protein, Nuc-Ploc predicted 7 proteins as chromatin and 2 as hetrochromatin binding protein, whereas Snlpred predicted all 11 proteins as chromatin binding protein. Out of 38 chromosomal proteins, SubNucPred correctly identified 16, Scp 1 and Nuc-Ploc predicted 2 proteins as chromatin binding respectively while Snlpred predicted 7 proteins as chromatin binding. Nuclear envelope proteins were predicted by other web-servers as nuclear lamina, which is not exactly identical to the nuclear envelope. Similarly in case of telomeric proteins, SubNucPred correctly predicted 2 telomeric proteins while Nuc-Ploc and Snlpred predicted 1 protein as chromatin and Scp did not provide any telomeric protein. The detailed prediction result is shown in Table S13.

In summary, considering the fact that there is difference in locations, we can't unambiguously conclude that performance of SubNucPred is inferior to the remaining three web-servers.

### Web-Server and Standalone Software

For the convenience of scientific community, a user-friendly web-server is also established at http://14.139.227.92/mkumar/subnucpred/, which may help in predicting the sub-nuclear location of nuclear proteins. This web-server can predict up to 25 sequences at a time and if a user gives more than 25 sequences, it will automatically predict only first 25 sequences. We also developed standalone version of SubNucPred, which can be installed locally and used for large-scale prediction. It can be downloaded from http://14.139.227.92/mkumar/subnucpred/download.html.

## Conclusions

We developed a combined method, SubNucPred, for predicting the sub-nuclear location of nuclear proteins with high accuracy by combining Pfam domain information and SVM score. We have also developed a publicly available web-server, which allows users to predict the sub-nuclear location of nuclear proteins. It is anticipated that the reported method may become a useful tool in speeding up the pace of nuclear proteins annotation. One of the shortcomings we see in our method that it works only for the known nuclear proteins and it will fail to classify in case a non-nuclear protein is submitted. The prediction rate of SubNucPred is expected to improve as more Pfam domains become available.

## Supporting Information

Figure S1 ROC curve of dipeptide composition based SVM modules.
(TIF)

Figure S2 ROC curve of physicochemical properties based SVM modules.
(TIF)

Table S1 Number of proteins present in different sub-nuclear locations in DataMAIN and DataIND.
(DOC)

Table S2 Number of single sub-nuclear location Pfam domains in different sub-nuclear locations.
(DOC)

Table S3 Unique Pfam domains present in different sub-nuclear locations. These domains are present only in a single sub-nuclear location nowhere else. Referred as single sub-nuclear location domain (SSLD) in the manuscript.
(DOC)

Table S4 Pfam domains which are present exclusively in two different sub-nuclear locations, nowhere else.
(DOC)

Table S5 30 physiochemical properties of amino acids selected from AAindex database to make SVM model (Source: Han et al (2013) PLoS One 8: e57225.).
(DOC)

Table S6 ANOVA test for analysis of difference in occurrence of different amino acids at P-value 0.01 (Table value  = 2.40, df1 = 9, df2 = 659).
(DOC)

Table S7 Performance of SVM models based on amino acid composition without using layer approach of training during LOOCV. (For detail please see Table S8).
(DOC)

Table S8 Performance of SVM model on amino acid composition without using layer approaches at different threshold. (during LOOCV).
(XLSX)

Table S9 Performance of SVM model based on amino acid composition using layer approach at different threshold (during LOOCV).
(XLSX)

Table S10 Performance of SVM model during LOOCV based on dipeptide composition using layer approach.
(DOC)

Table S11 Performance of SVM model during LOOCV based on physiochemical properties of amino acids using layer approaches.
(DOC)

Table S12 Prediction on the basis of Pfam domains using DataIND.
(DOC)

Table S13 Performance of SubNucPred web-server on DataIND.
(XLSX)
