# Supplementary figures and images for: Protein Sub-Nuclear Localization Prediction Using SVM and Pfam Domain Information
Source: PLoS One. 2014 Jun 4;9(6):e98345. doi: 10.1371/journal.pone.0098345 (PMC4045734; doi:10.1371/journal.pone.0098345)

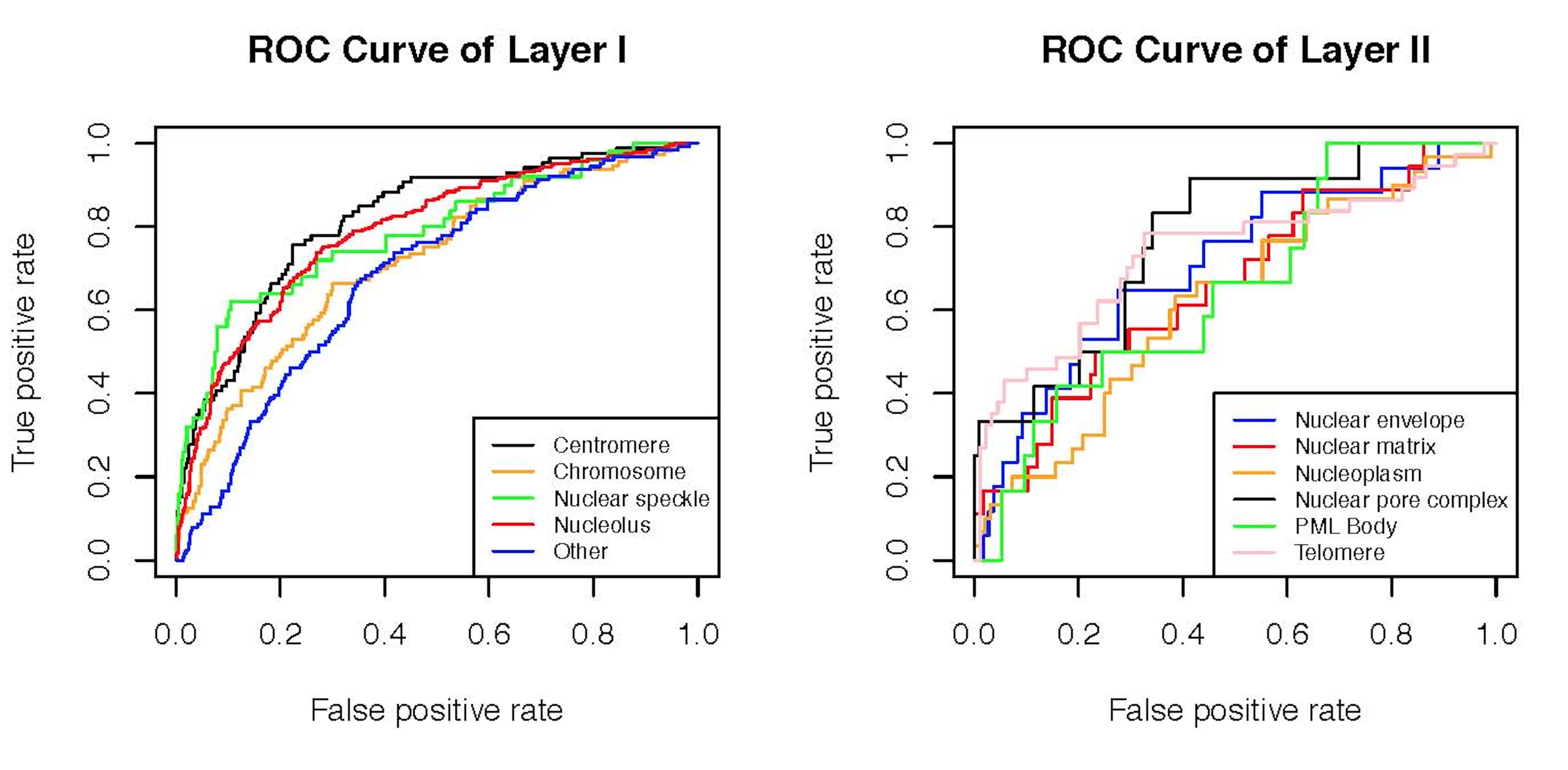

Supplement: Figure S1 — ROC curve of dipeptide composition based SVM modules. (TIF) [file pone.0098345.s001.tif]

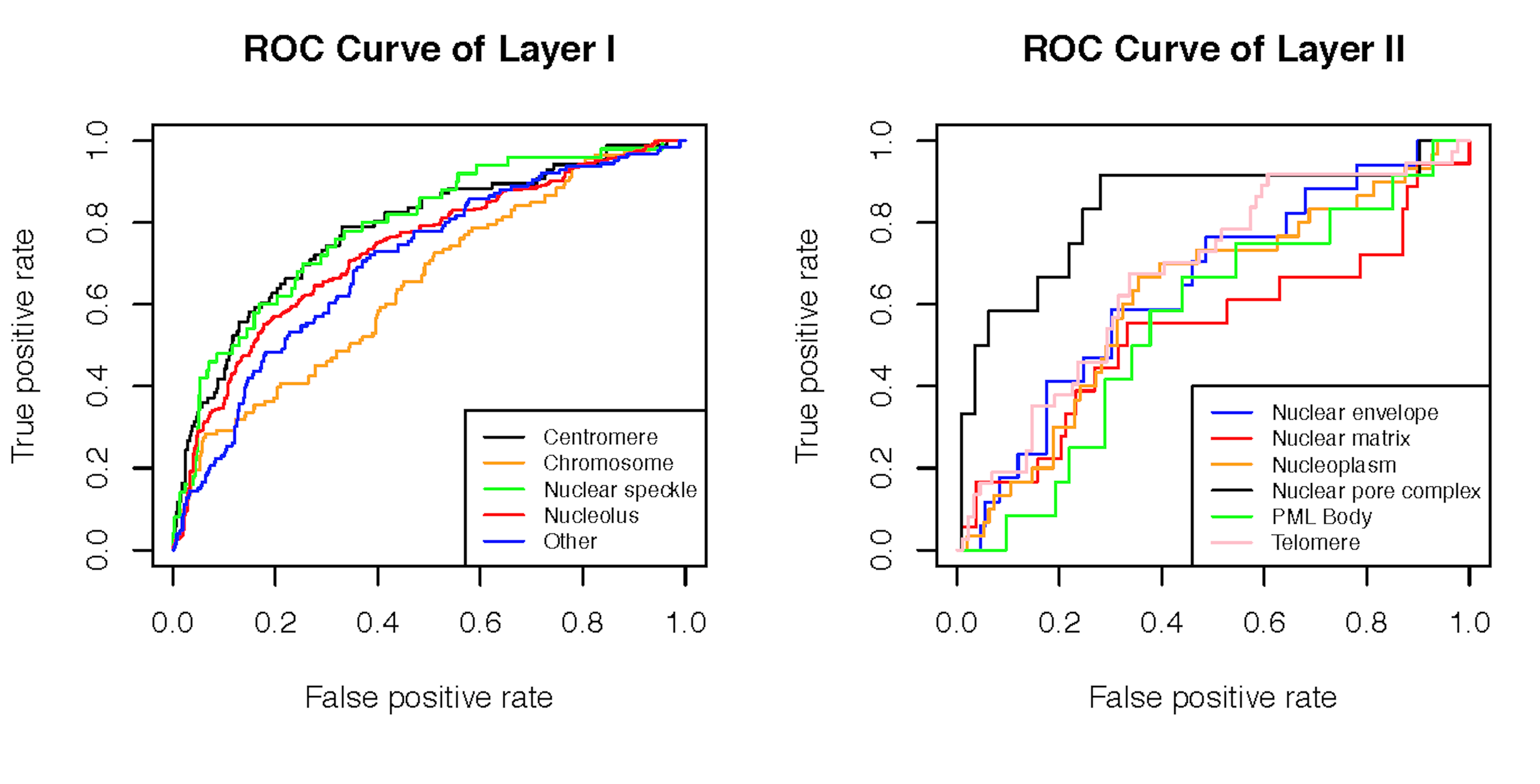

Supplement: Figure S2 — ROC curve of physicochemical properties based SVM modules. (TIF) [file pone.0098345.s002.tif]
