# Supplementary material for: Protein Sub-Nuclear Localization Prediction Using SVM and Pfam Domain Information
Source: PLoS One. 2014 Jun 4;9(6):e98345. doi: 10.1371/journal.pone.0098345 (PMC4045734; doi:10.1371/journal.pone.0098345)
Supplement: Table S1 — Number of proteins present in different sub-nuclear locations in DataMAIN and DataIND. (DOC) [file pone.0098345.s003.doc]

| Location | Data^MAIN^ | Data^IND^ |
| --- | --- | --- |
| Centromere | 86 | 31 |
| Chromosome | 113 | 38 |
| Nucleolus | 294 | 46 |
| Nuclear speckle | 50 | 14 |
| Telomere | 37 | 5 |
| Nucleoplasm | 30 | 7 |
| Nuclear pore complex | 12 | 2 |
| Nuclear matrix | 18 | 6 |
| Nuclear envelope | 17 | 51 |
| PML body | 12 | 7 |
| **Total** | **669** | **207** |
