# Supplementary material for: Protein Sub-Nuclear Localization Prediction Using SVM and Pfam Domain Information
Source: PLoS One. 2014 Jun 4;9(6):e98345. doi: 10.1371/journal.pone.0098345 (PMC4045734; doi:10.1371/journal.pone.0098345)
Supplement: Table S2 — Number of single sub-nuclear location Pfam domains in different sub-nuclear locations. (DOC) [file pone.0098345.s004.doc]

| **Location** | **Number of Domains** | |
| --- | --- | --- |
|  | **Before removing domains present in Non-nuclear Location** | **After removing domains present in Non-nuclear Locations** |
| Centromere | 32 | 21 |
| Chromosome | 43 | 19 |
| Nuclear speckle | 31 | 16 |
| Nucleolus | 201 | 81 |
| Nuclear envelope | 18 | 6 |
| Nuclear matrix | 7 | 3 |
| Nucleoplasm | 17 | 7 |
| Nuclear pore complex | 8 | 4 |
| PML body | 8 | 4 |
| Telomere | 19 | 10 |
| **Total** | **384** | **171** |
