# Supplementary material for: Protein Sub-Nuclear Localization Prediction Using SVM and Pfam Domain Information
Source: PLoS One. 2014 Jun 4;9(6):e98345. doi: 10.1371/journal.pone.0098345 (PMC4045734; doi:10.1371/journal.pone.0098345)
Supplement: Table S3 — Unique Pfam domains present in different sub-nuclear locations. These domains are present only in a single sub-nuclear location nowhere else. Referred as single sub-nuclear location domain (SSLD) in the manuscript. (DOC) [file pone.0098345.s005.doc]

| **Sr. No.** | **Domain Name** |
| --- | --- |
| 1. Centromere | |
|  | CENP-B_N |
|  | CENP-H |
|  | CENP-K |
|  | Cenp-B_dimeris |
|  | Cenp-O |
|  | Cnl2_NKP2 |
|  | Mis12 |
|  | Mis12_component |
|  | Mis14 |
|  | Mis6 |
|  | YDG_SRA |
|  | Ndc80_HEC |
|  | Nnf1 |
|  | Nuf2 |
|  | ORC2 |
|  | SANTA |
|  | Shugoshin_C |
|  | Shugoshin_N |
|  | Sir1 |
|  | Spc7 |
|  | Spindle_Spc25 |
|  |  |
| 1. Chromosome | |
|  | ASF1_hist_chap |
|  | COBRA1 |
|  | Cmyb_C |
|  | Cnd1_N |
|  | Cohesin_HEAT |
|  | DUF3639 |
|  | EZH2_WD-Binding |
|  | Med17 |
|  | Meiotic_rec114 |
|  | Mer2 |
|  | Nipped-B_C |
|  | Protamine_P1 |
|  | Protamine_P2 |
|  | SPT16 |
|  | SSrecog |
|  | Spt5_N |
|  | TH1 |
|  | TP1 |
|  | VEFS-Box |
|  |  |
| 1. Nucleolus | |
|  | ARID |
|  | BCAS2 |
|  | BOP1NT |
|  | BP28CT |
|  | CAF1C_H4-bd |
|  | CPL |
|  | Cgr1 |
|  | Clp1 |
|  | Cytokin_check_N |
|  | DBP10CT |
|  | DDT |
|  | DNA_RNApol_7kD |
|  | DTHCT |
|  | DUF1168 |
|  | DUF2361 |
|  | DUF3245 |
|  | DUF3321 |
|  | DUF3381 |
|  | DUF947 |
|  | EXOSC1 |
|  | zf-LYARe |
|  | Ebp2 |
|  | Fcf2 |
|  | GN3L_Grn1 |
|  | HDAC_interact |
|  | Helicase_Sgs1 |
|  | Las1 |
|  | Mak16 |
|  | Mpp10 |
|  | NGP1NT |
|  | NLE |
|  | NOC3p |
|  | NOGCT |
|  | NOP5NT |
|  | NUC129 |
|  | NUC130_3NT |
|  | NUC153 |
|  | NUC205 |
|  | Noc2 |
|  | Nop14 |
|  | Nop16 |
|  | Nop25 |
|  | Nop52 |
|  | Npa1 |
|  | Nrap |
|  | NuA4 |
|  | P120R |
|  | P19Arf_N |
|  | P68HR |
|  | PAH |
|  | PLU-1 |
|  | PMC2NT |
|  | POP1 |
|  | POPLD |
|  | RFC1 |
|  | RNA_polI_A14 |
|  | RNA_pol_I_TF |
|  | RNA_pol_Rpa2_4 |
|  | RNA_pol_Rpb8 |
|  | RRN3 |
|  | RRN7 |
|  | RRN9 |
|  | RRS1 |
|  | SDA1 |
|  | SURF6 |
|  | Sas10_Utp3_C |
|  | Sof1 |
|  | Spb1_C |
|  | Stc1 |
|  | TRAUB |
|  | Tat |
|  | U3_assoc_6 |
|  | U3_snoRNA_assoc |
|  | U3snoRNP10 |
|  | UAF_Rrn10 |
|  | UTP15_C |
|  | Urb2 |
|  | Utp11 |
|  | Utp13 |
|  | rRNA_processing |
|  | zf-C5HC2 |
| 1. Nuclear envelope | |
|  | B-block_TFIIIC |
|  | LBR_tudor |
|  | Ndc1_Nup |
|  | DUF1012 |
|  | NUC173 |
|  | Thymopoietin |
| 1. Nuclear matrix | |
|  | AKAP95 |
|  | EBV-NA3 |
|  | CUT |
| 1. Nuclear pore complex | |
|  | NUP50 |
|  | Nup88 |
|  | Nup153 |
|  | Nup_retrotrp_bd |
| 1. Nucleoplasm | |
|  | CDC73 |
|  | MMS1_N |
|  | YEATS |
|  | zf-RNPHF |
|  | CPSF_A |
|  | Paf1 |
|  | zf-C2HC |
| 1. Nuclear speckle | |
|  | AF-4 |
|  | EAF |
|  | MamL-1 |
|  | PRO8NT |
|  | PROCT |
|  | Pou |
|  | Transformer |
|  | U6-snRNA_bdg |
|  | DUF3437 |
|  | HAT |
|  | PHF5 |
|  | PROCN |
|  | PRP8_domainIV |
|  | RRM_4 |
|  | U5_2-snRNA_bdg |
|  | zf-U1 |
| 1. PML body | |
|  | CTNNB1_binding |
|  | Ets |
|  | Elf-1_N |
|  | Sp100 |
| 1. Telomere | |
|  | DUF1879 |
|  | N-Term_TEN |
|  | Rap1_C |
|  | TAN |
|  | TRF |
|  | Myb_DNA-bind_2 |
|  | Rap1-DNA-bind |
|  | Rif1_N |
|  | TEBP_beta |
|  | Telomere_Pot1 |
