# Supplementary material for: Protein Sub-Nuclear Localization Prediction Using SVM and Pfam Domain Information
Source: PLoS One. 2014 Jun 4;9(6):e98345. doi: 10.1371/journal.pone.0098345 (PMC4045734; doi:10.1371/journal.pone.0098345)
Supplement: Table S4 — Pfam domains which are present exclusively in two different sub-nuclear locations, nowhere else. (DOC) [file pone.0098345.s006.doc]

| **Location** | **Domain** |
| --- | --- |
| Centromere and Chromosome | CHL4, Pre-SET |
| Centromere and PML body | HIRA_B, Hira |
| Chromosome and Nucleoplasm | RRM_3 |
| Chromosome and Telomere | Ku_C |
| Nuclear envelope and Nuclear pore complex | Nic96 |
| Nuclear speckle and Nuclear matrix | Homeobox |
| Nuclear matrix and Nucleolus | HnRNP_M |
