# Supplementary material for: Protein Sub-Nuclear Localization Prediction Using SVM and Pfam Domain Information
Source: PLoS One. 2014 Jun 4;9(6):e98345. doi: 10.1371/journal.pone.0098345 (PMC4045734; doi:10.1371/journal.pone.0098345)
Supplement: Table S5 — 30 physiochemical properties of amino acids selected from AAindex database to make SVM model (Source: Han et al (2013) PLoS One 8: e57225.). (DOC) [file pone.0098345.s007.doc]

| **AAindex** | **Physicochemical property** | **Range of property** |
| --- | --- | --- |
| BULH740101 | Transfer free energy to surface | [-2.46 to 0.16] |
| BULH740102 | Apparent partial specific volume | [0.558 to 0.842] |
| PONP800102 | Average gain in surrounding hydrophobicity | [5.72 to 10.93] |
| PONP800104 | Surrounding hydrophobicity in alpha-helix | [10.98 to 15.36] |
| PONP800105 | Surrounding hydrophobicity in beta-sheet | [11.79 to 16.49] |
| PONP800106 | Surrounding hydrophobicity in turn | [9.93 to 15.00] |
| MANP780101 | Average surrounding hydrophobicity | [11.23 to 15.71] |
| EISD840101 | Consensus normalized hydrophobicity scale | [-1.76 to 0.73] |
| JOND750101 | Hydrophobicity | [0.00 to 3.77] |
| HOPT810101 | Hydrophilicity value | [-3.4 to 3.00] |
| PARJ860101 | HPLC parameter | [-10.00 to 10.00] |
| JANJ780101 | Average accessible surface area | [22.8 to 103.0] |
| PONP800107 | Accessibility reduction ratio | [1.79 to 7.69] |
| CHOC760102 | Residue accessible surface area in folded protein | [18 to 97] |
| ROSG850101 | Mean area buried on transfer | [62.9 to 224.6] |
| ROSG850102 | Mean fractional area loss | [0.52 to 0.91] |
| BHAR880101 | Average flexibility indices | [0.295 to 0.544] |
| KARP850101 | Flexibility parameter for no rigid neighbors | [0.925 to 1.169] |
| KARP850102 | Flexibility parameter for one rigid neighbor | [0.862 to 1.085] |
| KARP850103 | Flexibility parameter for two rigid neighbors | [0.803 to 1.057] |
| JANJ780102 | Percentage of buried residues | [3 to 74] |
| JANJ780103 | Percentage of exposed residues | [5 to 85] |
| LEVM780101 | Normalized frequency of alpha-helix, with weights | [0.52 to 1.47] |
| LEVM780102 | Normalized frequency of beta-sheet, with weights | [0.64 to 1.49] |
| LEVM780103 | Normalized frequency of reverse turn, with weights | [0.41 to 1.91] |
| GRAR740102 | Polarity | [4.9 to 13.0] |
| GRAR740103 | Volume | [3 to 170] |
| MCMT640101 | Refractivity | [0.00 to 42.35] |
| PONP800108 | Average number of surrounding residues | [4.88 to 7.86] |
| KYTJ820101 | Hydropathy index | [-4.5 to 4.5] |

^#^Han GS, Yu ZG, Anh V, Krishnajith AP, Tian YC (2013) An ensemble method for predicting subnuclear localizations from primary protein structures. PLoS One 8: e57225.
