# Supplementary material for: Protein Sub-Nuclear Localization Prediction Using SVM and Pfam Domain Information
Source: PLoS One. 2014 Jun 4;9(6):e98345. doi: 10.1371/journal.pone.0098345 (PMC4045734; doi:10.1371/journal.pone.0098345)
Supplement: Table S6 — ANOVA test for analysis of difference in occurrence of different amino acids at P-value 0.01 (Table value = 2.40, df1 = 9, df2 = 659). (DOC) [file pone.0098345.s008.doc]

| **Amino Acid** | **F-value** |
| --- | --- |
| A | 2.842628773 |
| C | 2.274160784 |
| D | 4.839730878 |
| E | 5.785295148 |
| F | 8.226599442 |
| G | 6.183734411 |
| H | 10.93767569 |
| I | 10.64801906 |
| K | 9.983691101 |
| L | 10.64801906 |
| M | 1.563625415 |
| N | 3.992173614 |
| P | 11.81879447 |
| Q | 3.924492794 |
| R | 3.366404841 |
| S | 8.637247537 |
| T | 51.74008999 |
| V | 4.73861982 |
| W | 3.210661315 |
| Y | 2.730391894 |

Values highlighted in red shows lower F-value than that of table value.
