# Supplementary material for: Protein Sub-Nuclear Localization Prediction Using SVM and Pfam Domain Information
Source: PLoS One. 2014 Jun 4;9(6):e98345. doi: 10.1371/journal.pone.0098345 (PMC4045734; doi:10.1371/journal.pone.0098345)
Supplement: Table S7 — Performance of SVM models based on amino acid composition without using layer approach of training during LOOCV. (For detail please see Table S8). (DOC) [file pone.0098345.s009.doc]

| **Location** | **TP** | **TN** | **FP** | **FN** | **Sensitivity** | **Specificity** | **Accuracy** | **MCC** |
| --- | --- | --- | --- | --- | --- | --- | --- | --- |
| Centromere | 67 | 524 | 159 | 19 | 77.91 | 76.72 | 76.85 | 0.38 |
| Chromosome | 76 | 423 | 233 | 37 | 67.26 | 64.48 | 64.89 | 0.23 |
| Nuclear speckle | 35 | 520 | 199 | 15 | 70.00 | 72.32 | 72.17 | 0.23 |
| Nucleolus | 211 | 346 | 129 | 83 | 71.77 | 72.84 | 72.43 | 0.44 |
| Nuclear envelope | 11 | 520 | 232 | 6 | 64.71 | 69.15 | 69.05 | 0.11 |
| Nuclear matrix | 12 | 508 | 243 | 6 | 66.67 | 67.64 | 67.62 | 0.11 |
| Nucleoplasm | 17 | 434 | 305 | 13 | 56.67 | 58.73 | 58.65 | 0.06 |
| Nuclear pore complex | 9 | 585 | 172 | 3 | 75.00 | 77.28 | 77.24 | 0.15 |
| PML body | 7 | 474 | 283 | 5 | 58.33 | 62.62 | 62.55 | 0.05 |
| Telomere | 25 | 499 | 233 | 12 | 67.57 | 68.17 | 68.14 | 0.16 |

Where TP, TN, FP, FN and MCC are True positive, True negative, False positive, False negative and Matthews correlation coefficient respectively.
