# Supplementary material for: Protein Sub-Nuclear Localization Prediction Using SVM and Pfam Domain Information
Source: PLoS One. 2014 Jun 4;9(6):e98345. doi: 10.1371/journal.pone.0098345 (PMC4045734; doi:10.1371/journal.pone.0098345)
Supplement: Table S10 — Performance of SVM model during LOOCV based on dipeptide composition using layer approach. (DOC) [file pone.0098345.s012.doc]

| **Location** | **TP** | **TN** | **FP** | **FN** | **Sensitivity** | **Specificity** | **Accuracy** | **MCC** | **AUC** |
| --- | --- | --- | --- | --- | --- | --- | --- | --- | --- |
| **Layer-I** | | | | | | | | |  |
| Centromere | 65 | 525 | 158 | 21 | 75.58 | 76.87 | 76.72 | 0.36 | 0.82 |
| Chromosome | 75 | 459 | 197 | 38 | 66.37 | 69.97 | 69.44 | 0.27 | 0.72 |
| Nuclear speckle | 36 | 519 | 200 | 14 | 72.00 | 72.18 | 72.17 | 0.24 | 0.79 |
| Nucleolus | 221 | 341 | 134 | 73 | 75.17 | 71.79 | 73.08 | 0.46 | 0.80 |
| Others | 85 | 415 | 228 | 41 | 67.46 | 64.54 | 65.02 | 0.24 | 0.68 |
| **Layer-II** | | | | | | | | |  |
| Nuclear envelope | 11 | 78 | 31 | 6 | 64.71 | 71.56 | 70.63 | 0.26 | 0.71 |
| Nuclear matrix | 11 | 64 | 44 | 7 | 61.11 | 59.26 | 59.52 | 0.14 | 0.66 |
| Nucleoplasm | 19 | 58 | 38 | 11 | 63.33 | 60.42 | 61.11 | 0.20 | 0.62 |
| Nuclear pore complex | 8 | 78 | 36 | 4 | 66.67 | 68.42 | 68.25 | 0.22 | 0.77 |
| PML body | 6 | 67 | 47 | 6 | 50.00 | 58.77 | 57.94 | 0.05 | 0.65 |
| Telomere | 26 | 62 | 27 | 11 | 70.27 | 69.66 | 69.84 | 0.37 | 0.74 |

Where TP, TN, FP, FN, MCC and AUC are True positive, True negative, False positive, False negative, Matthews correlation coefficient and area under ROC curve respectively.
