# Supplementary material for: Protein Sub-Nuclear Localization Prediction Using SVM and Pfam Domain Information
Source: PLoS One. 2014 Jun 4;9(6):e98345. doi: 10.1371/journal.pone.0098345 (PMC4045734; doi:10.1371/journal.pone.0098345)
Supplement: Table S11 — Performance of SVM model during LOOCV based on physiochemical properties of amino acids using layer approaches. (DOC) [file pone.0098345.s013.doc]

| **Location** | **TP** | **TN** | **FP** | **FN** | **Sensitivity** | **Specificity** | **Accuracy** | **MCC** | **AUC** |
| --- | --- | --- | --- | --- | --- | --- | --- | --- | --- |
| **Layer-I** | | | | | | | | |  |
| Centromere | 60 | 506 | 177 | 26 | 69.77 | 74.08 | 73.60 | 0.30 | 0.78 |
| Chromosome | 70 | 370 | 286 | 43 | 61.95 | 56.40 | 57.22 | 0.13 | 0.65 |
| Nuclear speckle | 35 | 532 | 187 | 15 | 70.00 | 73.99 | 73.73 | 0.24 | 0.79 |
| Nucleolus | 191 | 336 | 139 | 103 | 64.97 | 70.74 | 68.53 | 0.35 | 0.73 |
| Others | 86 | 416 | 227 | 40 | 68.25 | 64.70 | 65.28 | 0.25 | 0.70 |
| **Layer-II** | | | | | | | | | |
| Nuclear envelope | 10 | 62 | 47 | 7 | 58.82 | 56.88 | 57.14 | 0.11 | 0.64 |
| Nuclear matrix | 10 | 66 | 42 | 8 | 55.56 | 61.11 | 60.32 | 0.12 | 0.54 |
| Nucleoplasm | 20 | 61 | 35 | 10 | 66.67 | 63.54 | 64.29 | 0.26 | 0.62 |
| Nuclear pore complex | 9 | 89 | 25 | 3 | 75.00 | 78.07 | 77.78 | 0.35 | 0.83 |
| PML body | 7 | 70 | 44 | 5 | 58.33 | 61.40 | 61.11 | 0.12 | 0.56 |
| Telomere | 25 | 58 | 31 | 12 | 67.57 | 65.17 | 65.87 | 0.30 | 0.67 |

Where TP, TN, FP, FN, MCC and AUC are True positive, True negative, False positive, False negative, Matthews correlation coefficient and area under ROC curve respectively.
