# Supplementary material for: Protein Sub-Nuclear Localization Prediction Using SVM and Pfam Domain Information
Source: PLoS One. 2014 Jun 4;9(6):e98345. doi: 10.1371/journal.pone.0098345 (PMC4045734; doi:10.1371/journal.pone.0098345)
Supplement: Table S12 — Prediction on the basis of Pfam domains using DataIND. (DOC) [file pone.0098345.s014.doc]

| Actual Location | **Predicted Location** | | | | | | | | | | |
| --- | --- | --- | --- | --- | --- | --- | --- | --- | --- | --- | --- |
|  | Centromere | Chromo^#^ | Nucleolus | Nuclear speckle | Nuclear  envelope | Nuclear  Matrix | Nucleoplasm | NPC^&^ | PML^*^ | Telomere | No unique domain found |
| Centromere (31) | 1 |  |  |  |  |  |  |  |  |  | 30 |
| Chromosome (38) |  | 1 |  |  |  |  |  |  |  |  | 37 |
| Nuclear speckle (14) |  |  |  | 0 |  |  |  |  |  |  | 14 |
| Nucleolus (46) |  |  | 7 |  |  |  |  |  |  |  | 39 |
| Nuclear envelope (51) |  |  |  |  | 3 |  |  |  |  |  | 48 |
| Nuclear matrix (6) |  |  |  |  |  | 0 |  |  |  |  | 6 |
| Nucleoplasm (7) |  |  | 1 |  |  |  | 0 |  |  |  | 6 |
| Nuclear pore complex (2) |  |  |  |  |  |  |  | 0 |  |  | 2 |
| PML body (7) |  |  |  |  |  | 1 |  |  | 0 |  | 6 |
| Telomere (5) |  |  |  |  |  |  |  |  |  | 1 | 4 |

^#^Chromosome

^&^Nuclear pore complex

^*^PML body
